# Accommodative amplitudes and high-order aberrations in patients with multiple sclerosis with optic nerve ınvolvement

**DOI:** 10.5935/0004-2749.20210090

**Published:** 2025-08-21

**Authors:** Esra Vural, Deniz Kılıç, Ersin Kasım Ulusoy

**Affiliations:** 1 Department of Ophthalmology, Kayseri City Hospital, Kayseri, Turkey; 2 Department of Neurology, Kayseri City Hospital, Kayseri, Turkey

**Keywords:** Cornea, Accommodation ocular, Multiple sclerosis, Optic nerve, Córnea, Acomodação ocular, Esclerose múltipla, Nervo óptico

## Abstract

**Purpose:**

This study aimed to investigate and compare the changes in corneal aberrations and accommodative amplitudes between patients with multiple sclerosis and normal individuals.

**Methods:**

We included 20 patients who were previously diagnosed with multiple sclerosis with optic nerve involvement (multiple sclerosis group) and 20 healthy sexand age-matched individuals (control group). We only selected those who were under 40 years old because accommodation in individuals over 40 years old significantly deteriorates. We measured the accommodative amplitude in diopters by minus lens test and evaluated the higher-order aberrations by using the iDesign aberrometer. Then, we compared the accommodative amplitude and the root mean square of higher-order aberrations between the groups.

**Results:**

The mean age of the multiple sclerosis and control groups were 35.25 ± 4.52 and 32.28 ± 6.83 years, respectively (*p*=0.170). The accommodative amplitude was 4.05 ± 1.25 D in the multiple sclerosis group and 6.00 ± 1.03 D in the control group, with a statistically significant difference (*p*<0.001). Meanwhile, the root mean square of higher-order aberrations was not significantly different between the groups (multiple sclerosis group, 0.44 ± 0.22; control group, 0.43 ± 0.10, *p*<0.824). Moreover, aberration changes had no statistically significant differences between the two groups at baseline and at 5 D stimulus.

**Conclusions:**

The accommodative amplitude was decreased in patients with multiple sclerosis, suggesting the possible cause of transient visual impairments in these patients. However, this accommodative amplitude did not demonstrate a significant difference in terms of higher-order aberration change during accommodation between such patients and the controls.

## Abstract (pt)

**Objetivo:**

Investigar se as aberrações da córnea e as amplitudes de acomodação alteram mais em pacientes com esclerose múltipla do que em populações normais.

**Métodos:**

Vinte pacientes previamente diagnosticados com esclerose múltipla com envolvimento do nervo óptico (grupo com eslerose múltipla) e 20 indivíduos saudáveis pareados por sexo e idade (grupo controle) foram incluídos no estudo. Pacientes com menos de 40 anos de idade foram incluídos em ambos os grupos devido à deterioração significativa de acomodação em pacientes com mais de 40 anos de idade. Para cada participante, a amplitude de acomodação foi medida em dioptrias pelo teste de lentes negativas e as aberrações de alta ordem foram avaliadas com o aberrômetro iDesign. Em seguida, a amplitude de acomodação e a média da raiz quadrada de aberrações de alta ordem foram comparadas entre os grupos.

**Resultados:**

As médias da idade dos grupos com esclerose múltipla e controle foram 35,25 ± 4,52 anos e 32,28 ± 6,83 anos, respectivamente (p=0,170). A amplitude de acomodação foi de 4,05 ± 1,25 D no grupo com esclerose múltipla e 6,00 ± 1,03 D no grupo controle. A diferença entre os com esclerose múltipla e o grupo controle foi estatisticamente significativa (p<0, 001). A média da raiz quadrada das aberrações de alta ordem não foi significativamente diferente entre os grupos (com esclerose múltipla, 0,44 ± 0,22; controle, 0,43 ± 0,10, p<0,824). Não houve diferenças estatisticamente significativas entre os grupos em termos de alterações de aberrações entre a linha de base e o estímulo 5 D.

**Conclusões:**

Este estudo mostra que a amplitude de acomodação diminuiu em pacientes com esclerose múltipla. Portanto, esses resultados podem causar possíveis razões de deficiências visuais transitórias em pacientes com esclerose múltipla. No entanto, esta diferença de amplitude de acomodação não fez uma diferença significativa entre os grupos quanto à alteração das aberrações de alta ordem durante a acomodação.

## INTRODUCTION

Multiple sclerosis (MS) is an inflammatory demyelinating disease characterized by neuroaxonal degeneration at multiple sites of the central nervous system (CNS)^(1,2)^. Its etiology remains uncertain, but it is reportedly caused by an autoimmune reaction against CNS-specific myelin and myelin-forming oligodendrocytes^(3)^. In MS, axonal damages occur throughout the CNS, and the clinical manifestations vary with the affected neurons, which are frequently the visual pathway neurons^(3,4)^.

Visual impairment is one of the most common symptoms of MS; approximately 80% of patients complain of visual deterioration during the disease course^(4,5)^. Both the afferent and efferent pathways of the visual system are disrupted, and ocular findings are associated with the disruption of these pathways^(6)^. The most common ocular finding is optic neuritis, which is characterized by a damaged afferent pathway^(5)^. Disorders involving the efferent pathways occur as ocular motor deficits, which include internuclear ophthalmoplegia and nystagmus^(7,8)^.

Accommodation refers to the adjustment of the dioptric power of the eye to obtain a clear retinal image as the distance changes^(9)^. It is a complex response involving the afferent (from retina and optic nerve to the occipital lobe) and efferent pathways (the oculomotor nerve innervating the sphincter of the pupilla and the ciliary muscles)^(10)^. The accommodation is mainly controlled by parasympathetic innervation of the ciliary muscle; meanwhile, the sympathetic innervation plays a role in the relaxing phase of accommodation^(11)^.

The maximum accommodative capacity of the eye is defined as accommodative amplitude (AA)^(12)^. The AA decreases especially with aging (presbyopia), but it may also decrease in individuals with primary ocular diseases or systemic or neurological disorders^(13)^.

Higher-order aberrations (HOAs) are complex and subtle refractive errors occurring in any part of the optical axis, having an impact on visual and retinal image quality of an eye. The HOAs depend on age, refractive error (myopia, hypermetropia, or astigmatism), and pupil size, as well as tear film instability and corneal irregularities^(14)^. Accommodation may alter the HOAs in healthy individuals^(15)^.

We hypothesized that the AA might decrease as a result of neurological impairment in patients with MS. This study aimed to assess the changes in AA and HOAs during accommodation in patients with MS. To our knowledge, our study is the first to assess the HOAs during accommodation in patients with MS.

## METHODS

### Study Design and Population

This prospective, case-control study was conducted at Research and Educational Hospital between October 2018 and February 2019. Its methodology was approved by the Institutional Review Board, and the research protocol adhered to the tenets of the Declaration of Helsinki for clinical research. A written informed consent was obtained from each participant after being explained on the purpose and possible consequences of the study. We included 20 eyes of 20 patients, which were previously diagnosed with MS with optic nerve involvement by the Neurology and Ophthalmology Department, (MS group) and the right eyes of 20 ageand sex-matched healthy subjects (control group). The optic nerve involvement in patients with MS was confirmed by detailed ophthalmological examination (best corrected visual acuity [BCVA], anterior and posterior segment examination, and direct and indirect light reflexes), retinal nerve fiber analysis, and visual field test. All of the included MS patients had optic nerve involvement findings in visually evoked potential (VEP) examination. Considering the significant deterioration of accommodation over the age of 40 years, we only selected individuals who were under 40 years old (25-40 years) in both groups. The exclusion criteria were as follows: patients with BCVA <20/20, cylindrical and spherical refractive errors >2 diopters, smoking, history of optic neuritis in the last 6 months, concomitant systemic diseases (diabetes, hypertension, renal dysfunction, or hepatic dysfunction), current systemic or ocular medical therapies, and any other ocular pathologies (e.g., glaucoma, retinal disease, and corneal opacity), and strabismus or other extraocular muscle involvement.

All patients underwent a detailed ophthalmological examination including BCVA measurement (Snellen charts), intraocular pressure measurement with a Goldmann applanation tonometer (Haag-Streit Inc., Köniz, Switzerland), and anterior segment evaluation by slit-lamp biomicroscopy and fundus examination from dilated pupilla by slit-lamp biomicroscopy with +90 D lens.

### Measurement of AA

The same experienced ophthalmologist (EV), who was blinded to the study subjects, conducted all the AA measurements using the spherical lens test, specifically at 9-11 AM to overcome the bias of the diurnal change in accommodation^(16)^. Initially, all the participants were examined for distance visual acuity. After BCVA determination, the participant focused on a stationary target while the AA was measured using plus or minus lenses. The participants were asked to read the N8 target at 40 cm distance. Plus lenses were added until the print was blurred, and then minus spheres were gradually added until the print was blurred again. The differences between plus and minus lenses is the AA.

### Evaluation of the Aberrations

The aberrations of the participants were assessed using the new-generation Hartmann-Shack aberrometer (iDesign aberrometer, Abbott Medical Optics, Abbott Park, Chicago, IL, USA). We also recorded the root mean square (RMS) of the total HOAs; spherical, vertical, and horizontal comas; and trefoil aberration at baseline and at 5 D stimulus.

### Statistical analysis

Statistical data were analyzed by the Statistical Package for the Social Sciences (SPSS^®^) 24.0 version on a Windows^®^-based PC. Descriptive statistics were expressed as “mean ± standard deviation (SD),” “frequency,” and “ratio.” The distribution of variables was evaluated by the Kolmogorov-Smirnov test. Quantitative values were compared between the two groups by using the *t*-test and Mann-Whitney *U* test. For comparing the qualitative variables, we used the χ^*2*^ test. Correlation analysis was performed using Pearson correlation coeffi cients. A p-value <0.05 was considered to be statistically significant.

## RESULTS

The mean ages of the MS group and the control group were 35.25 ± 4.52 and 32.28 ± 6.83 years, respectively; the difference was not statistically significant (*p*=0.17). The MS group consisted of 10 males and 10 females, whereas the control group had 11 males and 9 females. The AA in the MS group was 4.05 ± 1.25 D, whereas that in the control group was 6.00 ± 1.03 D, exhibiting a significant difference (*p*<0.001). However, the RMS results for the HOAs between the MS and control groups had no significant difference (*p*=0.824); differences in the HOA values between at baseline and at 5 D stimulus in both groups are shown in table 1. The aberration changes had no statistically significant differences between the two groups. Furthermore, no significant correlations were found between the HOA difference at baseline or at 5 D stimulus and AA (Table 2).

**Table 1:** Difference in higher-order aberrations at baseline and at 5 D stimulus between the two groups

Aberration	MS group	Control group	p-value
Spherical	-0.139 ± 0.085	-0.159 ± 0.029	0.352
Vertical coma	-0.013 ± 0.060	-0.040 ± 0.042	0.115
Horizontal coma	0.023 ± 0.033	0.001 ± 0.042	0.096
Trefoil	0.018 ± 0.075	0.042 ± 0.062	0.292
Total RMS	0.44 ± 0.22	0.43 ± 0.10	0.824

Data are means ± SD; *p*<0.05 was considered statistically significant between the MS group and the control group (independent t-test).

**Table 2:** Correlation between the change in higher-order aberrations at baseline and at 5 D stimulus, and accommodation amplitudes

	Spherical	Vertical coma	Horizontal coma Trefoil	RMS
Accommodation amplitude	*p*=0.682	*p*=0.337	*p*=0.474 *p*=0.192	*p*=0.238
	r=0.069	r=-0.160	r=-0.120 r=0.216	r=-0.199

* Pearson correlation analysis was used.

Our study focused on whether accommodation has an effect on demyelinating diseases such as MS, and we demonstrated that AA was decreased in patients with MS with ocular involvement, but HOA did not exhibit any effect.

Accommodation involves afferent and efferent pathways. When the target, which the eye was previously fixated, was placed anteriorly, the stimulus occurs in the retina. The visual impulse travels to the retinal ganglion cells and then to the visual cortex by the optic nerve, optic chiasma, optic tract, and optic radiation. In the midbrain, the afferent fibers synapse with the oculomotor nucleus and the Edinger-Westphal (EW) nucleus^(9-16)^. The oculomotor nerve carries the motor fibers to both medial rectus muscles to converge. The fibers emerging from the parasympathetic autonomic nucleus (EW nucleus) spread through the oculomotor nerve and synapse in the ciliary ganglion^(17)^. Postganglionic fibers named as the short ciliary nerves innervate the sphincter pupillae muscle and the ciliary muscle^(18)^. The arrival of impulses causes an increase in the dioptric power of the eye. The commonly accepted theories in the mechanism of such an increase are the Helmholtz and Schachar theories^(19,20)^. According to the Helmhotz theory, the accommodation begins with the circumferential contraction of the ciliary muscle, releasing the tension on the zonules^(20)^. The reduced zonular tension leads to several modifications in the lens capsule as well as in the equatorial lens diameter. However, the Schachar theory states that the accommodation begins with the contraction of radial fibers. This contraction increases the equatorial zonule tension, thereby increasing the lens diameter. Next, the circular fibers of the ciliary muscle contract, and the anterior and posterior zonules subsequently relax. The convexity of the central part of the lens increases, while the peripheral portion flattens. The increase in the surface curvature of the lens leads to the increase in the optical power^(19)^. Although the two theories are different, they both postulated that the intrinsic muscles of the eye control the accommodation. The autonomic nervous system regulates and controls these complex and remarkable afferent and efferent pathways of accommodation^(17)^.

The mechanism of dysregulation of the autonomic nervous system in patients with MS remains unclear^(21,22)^. Although the major visual symptoms in patients with MS (light sensitivity, insufficient color discrimination, and blurred vision) are mainly caused by the optic nerve involvement, visual impairment such as reading difficulty may result from the dysfunctions of the autonomic nervous system; this symptom might be the first and only clinical symptom in MS^(6,23)^. Thus, we aimed to evaluate the change in AA.

The assessment of best corrected distance visual acuity and high-contrast visual acuity (HCVA) insufficiently detects visual impairment in patients with MS^(24)^. If available, low-contrast visual acuity (LCVA) measurement and color vision assessment should be performed because they are more sensitive parameters of vision disturbances^(25)^. The assessment of the best corrected near visual acuity is commonly skipped and is not considered as a routine test for patients with MS.

In clinical practice, the pupil size, visual field examinations, VEP amplitude assessment, and optical coherence tomography (OCT) imaging of both retinal layers and the optic disc head are widely used to further investigate patients with MS^(7)^. Although these tests provide reliable results of the disease, the patients can still experience unexplained visual problems^(23,24)^, which might be additionally caused by accommodation insufficiency and the HOAs. Therefore, we investigated whether deterioration in AA, which is an important physiological mechanism in reading ability, exists or whether the HOAs differ in patients with MS compared with those in normal individuals.

We found a significantly decreased AA in the MS group compared with that in the control group. Similarly, Kucuk et al. recently found a decreased AA in patients with MS. They also found a significant correlation between AA and positive VEP findings^(12)^. Our study is similar to Kucuk et al.’s study according to VEP findings, considering that we included patients with reduced AA and with prolonged latency in VEP tests.

Higher-order wavefront aberrations also change as the accommodation increases^(26)^. Zhou et al. showed that a gradient of increasing accommodation stimuli with 0.5% phenylephrine drops changed the wavefront aberration. They postulated that during accommodation, miosis causes interference. However, this interference disappears when the pupil is dilated; thus, all changes in optical aberrations were attributed to the changes in the contour of the crystalline lens^(27)^. In our study, accommodation affected the HOAs, consistent with previous studies, but no significant correlation was found between the HOA difference at baseline and at 5 D stimulus and AA. HOAs, especially coma and spherical aberration (SA), affect the image quality of the eye^(28)^. A negative SA is the combination leading to relatively low means a decreased contrast in the defocused retinal image. We found that SA shifted to negative when accommodative stimuli gradually increased, but no statistical differences were found between the two groups. However, the change in HOAs was also not significantly different at baseline and at 5 D stimulus between such groups. HOAs reportedly changes with age and accommodation^(29)^. Considering the confounding effect of accommodation, Zhang et al. used 1% tropicamide drops to compare the HOAs between children and adolescents^(30)^. They found that the RMS values and aberrations were different in different age groups^(30)^. Hence, ageand sex-matched normal controls were included in our study.

Zhou et al. further found an increase in the RMS of the total HOAs with increased accommodation in the healthy population^(27)^. In our study, no significant difference in the RMS of HOAs was found between the two groups when the eye was in a non-accommodative phase and in the 5 D accommodative stimuli. Considering the similarity of both groups during the non-accommodative phase, we perceive that the impaired AA, not the aberrations of the eye, causes the difference in HOAs between the two groups. Nevertheless, we believe that both the decreased AA and the aberrations of the eye affect the visual quality in patients with MS, especially in near works.

Meanwhile, our study has some limitations. Our sample size is small, and we did not evaluate the OCT findings, which could demonstrate the afferent pathway pathologies. Instead, of OCT, VEP abnormalities were considered as afferent pathologies.

In conclusion, although previous studies demonstrated that the aberrations change during accommodation in normal individuals, this study evaluated that both the AA and the aberrations change during accommodation in patients with MS. The AA of the MS group was significantly increased, but the change in aberrations during accommodation between the two groups was not statistically significant different. Therefore, during accommodation (e.g., near work), visual quality dısturbance may not occur because of the absence of a significant change in HOAs.
